# Hypertension control and its association with left ventricular hypertrophy in children and young people on dialysis: a longitudinal study

**DOI:** 10.1007/s00467-026-07351-1

**Published:** 2026-05-23

**Authors:** Gordon Bruce, Ravsimrat Chawla, Alexandra Savis, Hannah Samuels, Joanna Newton, John M. Simpson, Manish D. Sinha

**Affiliations:** 1 Department of Paediatric Nephrology, 3rd Floor Beckett House, Evelina London Children’s Hospital, Guys & St Thomas’ NHS Foundation Trust, Westminster Bridge Road, London, SE1 7EH UK; 2 Department of Clinical Pharmacology, School of Cardiovascular and Metabolic Medicine, King’s College London, London, UK

**Keywords:** Children, Young people, Kidney failure, Cardiovascular disease, Hypertension, Left ventricular hypertrophy, Left ventricular mass index, NT pro-BNP

## Abstract

**Background:**

Hypertension remains one of the most prevalent modifiable risk associations for adverse outcomes in children receiving dialysis. Despite this, limited longitudinal data exist regarding ambulatory blood pressure monitoring (ABPM) and cardiac outcomes in children and young people (CYP) initiating dialysis.

**Methods:**

This is a retrospective, longitudinal study of CYP age 5–18 with paired echocardiogram and ABPM evaluations over 12 months (“baseline” to “follow-up”) following dialysis initiation. Left ventricular hypertrophy (LVH) was defined by age-specific indexed left ventricular mass (LVMI) and LV mass-for-height z-score ≥ 95th percentile. Hypertension was defined if day, night, or 24-h ABPM ≥ 95th percentile and CYP was categorised as having persistent hypertension, resolved hypertension, persistent normotension, or emergent hypertension.

**Results:**

There were 32 CYP, with a mean age of 12.7 ± 3.1 years. LVH was present in 37.5% at baseline and 25% at follow-up. LVH rates improved on haemodialysis (baseline 52.9%, follow-up 17.6%, *P* = 0.031), but not on peritoneal dialysis. Higher rates of hypertension were found in LVH versus no LVH at follow-up. Masked hypertension rates was present in 14 (43.8%) at both baseline and follow-up. Mean LVMI was higher in persistently hypertensive versus persistently normotensive CYP at follow-up. ΔLVMI was independently associated with LVH at baseline (yes/no) (*β* = −0.482, *P* = 0.001), and Δlog N-terminal pro-brain b-type natriuretic peptide (NT Pro-BNP) (*β* = 0.412, *P* = 0.005).

**Conclusions:**

CYP with persistent hypertension had worst rates of LVH and indexed LV mass. A correlation between changes in NT Pro-BNP and LVMI highlights a probable role of fluid overload. Further research into using NT Pro-BNP as a biomarker to improve hypertension management is warranted.

**Graphical abstract:**

**Figure:**
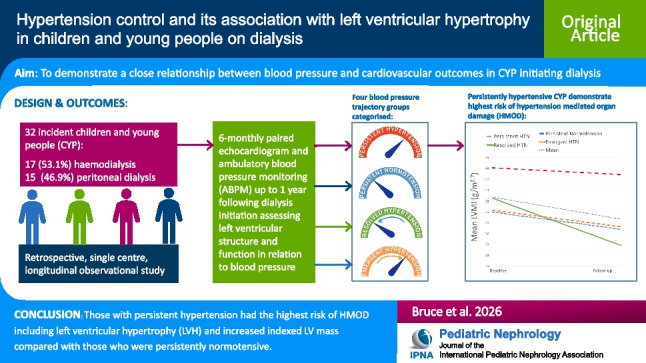
A higher resolution version of the Graphical abstract is available as Supplementary information

**Supplementary Information:**

The online version contains supplementary material available at 10.1007/s00467-026-07351-1.

## Introduction

Cardiovascular disease remains a leading cause of morbidity and mortality in children and young people (CYP) with kidney failure [1, 2]. Hypertension and left ventricular hypertrophy (LVH) are highly prevalent in CYP on dialysis, with rates as high as 80% and 85%, respectively [2–9]. LVH is the most common reported surrogate marker of adverse cardiovascular remodelling reflecting hypertension-mediated organ damage (HMOD) [10, 11].

While an association between hypertension and cardiovascular mortality in CYP is not clearly elucidated, robust registry data identifies uncontrolled hypertension as one of the strongest risk factors for LVH [12]. Previous studies highlight that improved blood pressure (BP) control reduces left ventricular mass (LVM), lowering rates of LVH [5, 13]. Optimal hypertension management therefore remains key to preventing adverse cardiovascular outcomes.

Accurate blood pressure measurement in CYP remains a challenge. Twenty-four-hour ambulatory blood pressure monitoring (ABPM) has been shown to precisely diagnose hypertension and improve its management and is thus considered gold standard [13–15]. Yet despite numerous studies reporting high rates of masked hypertension in CYP receiving dialysis, ABPM remains underutilized [16].

Few longitudinal studies in CYP on dialysis have evaluated a BP threshold that associates with improved markers of HMOD. In this report, our objective was to evaluate longitudinal changes in left ventricular structure and function with BP in a cohort of children initiating haemodialysis or peritoneal dialysis.

## Methods

### Study population

Retrospective, single-centre, longitudinal observational study including all incident consecutive CYP commencing in-centre intermittent haemodialysis (IHD) or receiving overnight continuous cycling peritoneal dialysis (CCPD) over a recent 3-year period.

### Inclusion and exclusion criteria

All CYP (i) who were aged 5–18 years and (ii) who commenced dialysis within the study period with at least two paired 24-h ABPM and echocardiogram studies, at dialysis start (baseline) and ≥ 6 months up to 1 year following dialysis initiation, were included. Exclusion criteria were (i) those younger than 5 years due to lack of normative ABPM data, (ii) those with congenital or structural heart disease, or (iii) those unable to tolerate investigation.

### Blood pressure measurement

ABPM monitoring was undertaken at every 6 month interval in CYP undergoing dialysis, and performed following mid-week dialysis if on IHD and following a clinic appointment on CCPD. Those on IHD typically had three 4-h sessions weekly. Those on CCPD received overnight therapy 7 days a week. Paired assessments (ABPM and echocardiography) were performed at baseline and 6-month intervals. In those with more than two assessments, we included the first and the last study evaluations and excluded the interim data for this study analysis. During ABPM, measurements were performed every 30 min through 24 h. Standardised auscultatory office BP was measured thrice using an aneroid sphygmomanometer at clinic.

Hypertension was defined if ABPM reported an average systolic, mean, or diastolic blood pressure ≥ 95th percentile (≥ 1.645 z-score) in 24 h, awake or sleep periods. Hypertension on office BP was categorised by systolic or diastolic ≥ 95th percentile, and masked hypertension was defined if normotensive on office BP and hypertensive on ABPM. In those > 16 years, threshold values as per 2016 European Society of Hypertension (ESH) guidelines were used [17].

Patients were categorised into four BP groups by comparing the first paired assessment (“baseline”) to final assessment (“follow-up”): (i) persistent hypertension, those with abnormal BP at both time points; (ii) resolved hypertension, those with hypertension at baseline and normotension at final assessment; (iii) persistent normotension, those with normotension at both time points; and (iv) emergent hypertension, those with newly occurred hypertension, those with normotension at baseline and hypertension at follow-up.

### Echocardiographic assessments

Echocardiography was performed on the same day as ABPM and at 6 month intervals. All studies were interpreted by one experienced paediatric cardiac sonographer (AS) performed within routine care. LVH was defined if ≥ 95th percentile (≥ 1.645 z-score) using (i) age-specific reference interval indexed LV mass (LVMI in g/m^2.7^) [18] and (ii) LV mass-for-height z-score [19]. Systolic dysfunction was assessed by left ventricular fractional shortening (FS), with < 25% indicating dysfunction. Mitral annulus diastolic motion (e’) was recorded using tissue Doppler imaging (TDi) at the basal mitral annulus (lateral and septal). Average e’ was calculated as (lateral e’ + septal e’)/2. To assess changes in diastolic function, left ventricular filling pressure was estimated by mitral valve E/average e’, the ratio of early LV filling velocity to early diastolic peak mitral annular velocity. E/e’ > 14 was reported as diastolic dysfunction and considered to be clinically significant, < 8 as normal, and between 8 and 14 as of “indeterminate clinical relevance” in keeping with previous reports in childhood chronic kidney disease (CKD) [11, 20].

### Interdialytic weight gain and biochemical parameters

Inter-dialysis weight difference (IDWD) was calculated by subtracting pre-dialysis weight from previous HD session post-dialysis weight and expressed as a percentage of the previous post-dialysis weight (IDWD%). Mean IDWD values were calculated using weekly readings in the month prior to ABPM.

Parathyroid hormone (PTH), 25-hydroxy vitamin D, and N-terminal pro-brain b-type natriuretic peptide (NT Pro-BNP) concentrations within 4 weeks of echocardiography were collated. NT Pro-BNP values were log-transformed to be normally distributed. Pre-HD phosphate, calcium, albumin, creatinine, and post-HD haemoglobin levels were evaluated as a mean of weekly measurements in the month prior to ABPM. In those on PD, they were measured at echocardiography.

### Statistical analysis

Findings are summarised as mean ± SD or median and interquartile range (IQR) if not normally distributed. Student’s *t*-test for independent samples, chi-squared test, or Fisher’s exact test was used for unpaired data. Student’s *t*-test for paired samples or McNemar’s test was used for paired data.

Univariate and backward stepwise multivariable regression analysis was used with ΔLVMI as the dependent variable. Independent variables included sex, age at commencement of dialysis, presence of LVH at baseline, prescribed anti-hypertensives at baseline, Δ 24-h mean arterial pressure (MAP), Δ haemoglobin, and Δ Log_10_ NT Pro-BNP.

In all analyses, statistical significance was established at *P* < 0.05. All tests were two-tailed. SPSS statistics software (IBM Corp. Released 2020. IBM SPSS Statistics for Macintosh, Version 28.0. Armonk, NY: IBM Corp) was used.

## Results

We included 32 CYP with a mean ± SD age of 12.7 ± 3.1 years. Demographic characteristics are summarised in Table 1. Seventeen patients (53.1%) commenced IHD, with 15 (46.9%) initiating CCPD. There was a median interval of 11 months between assessments. Twenty-one CYP (65.6%) had paired ABPM and echocardiogram studies scheduled 1 year apart between baseline and follow-up and 11 (34.4%) had studies at a 6-month interval due to later dialysis initiation.

**Table 1:** Demographic and clinical characteristics of *n* = 32 children and young people

Age at first ABPM	12.7 ± 3.1
Male sex *n* (%)	18 (56.3%)
Ethnicity *n* (%)	
White	13 (40.6%)
Black	5 (15.6%)
Southeast Asian	8 (25.0%)
Other	6 (18.7%)
Dialysis modality *n* (%)	
Haemodialysis	17 (53.1%)
Peritoneal dialysis	15 (46.9%)
Cause of kidney failure *n* (%)	
Glomerular	11 (34.4%)
CAKUT	11 (34.4%)
Polycystic kidney disease	2 (6.2%)
Ciliopathy	6 (18.8%)
Tubulointerstitial nephritis	2 (6.2%)

### Prevalence of hypertension and LVH at baseline and follow-up

Hypertension was present in 19 (59.4%) CYP at baseline and follow-up. Masked hypertension was present in 14 (43.8%) at both baseline and follow-up. In all patients, there was no significant change in mean ± SD BP between baseline and follow-up, with office SBP 114 ± 14 mmHg vs. 112 ± 14 mmHg (*P* = 0.45) and ABPM 24-h MAP 92 ± 14 mmHg vs. 90 ± 12 mmHg (*P *= 0.51).

The prevalence of LVH by age-specific indexed LV mass was 37.5% at baseline and 25% at follow-up (*P* = 0.39); using LV mass-for-height z-scores, the prevalence was 12.5% at both baseline and follow-up. Mean ± SD LVMI was 40.2 ± 13.2 vs. 37.2 ± 14.0 g/m^2.7^ (*P* = 0.12); and LV mass-for-height z-score was 0.31 ± 1.49 vs. −0.05 ± 1.55 (*P* = 0.12) at baseline and follow-up, respectively. The median (range) change in LVMI from baseline to follow-up (ΔLVMI) was −2.8 (−7.2, 2.2) g/m^2.7^, and ΔLV mass-for-height z-score −0.4 (−1.2, 0.4).

Of 19 hypertensive patients at baseline, 10 (52.6%) were hypertensive day and night, 2 (10.5%) were hypertensive during daytime, and 7 had isolated nocturnal hypertension (36.8%). At follow-up, 7 (36.8%) were hypertensive day and night and 12 (63.2%) had isolated nocturnal hypertension.

### Changes in BP and LV structure by dialysis modality

At baseline, 12/17 (70.6%) CYP on IHD were hypertensive, and 7/15 (46.7%) on CCPD were hypertensive. At follow-up, 9/17 (52.9%) on IHD and 10/15 (66.7%) on CCPD were hypertensive. Nine CYP (52.9%) initiating IHD had LVH by LVMI at baseline versus 3 (17.6%) at follow-up (*P* = 0.031). Three (20%) initiating CCPD had LVH at baseline vs. 5 (33.3%) at follow-up (*P* = 0.68); using LV mass-for-height z-score to define LVH, rates were lower (in those on IHD at baseline 4/17 (23.5%) versus at follow-up 3/17 (17.6%) (*P* = 1.00); in those on CCPD at baseline 0/15 (0%) versus at follow-up 1/15 (6.7%) (*P* = 1.00)). Further comparison by dialysis modality is outlined in Supplementary Table 1.

Twelve of 32 (37.5%) CYP had an increase in indexed LV mass between baseline and follow-up (IHD *n* = 7, CCPD *n* = 5). The median (range) ΔLVMI in this group was 5.1 (2.1–8.8) g/m^2.7^. In those on IHD, the mean ± SD LVMI was 43.7 ± 16.2 vs. 37.8 ± 17.4 g/m^2.7^ (*P* = 0.31), and LV mass-for-height z-score was 0.61 ± 1.73 vs. −0.08 ± 1.77 (*P* = 0.25) at baseline and follow-up. On CCPD, the mean ± SD LVMI was 36.1 ± 7.5 vs. 36.5 ± 9.5 g/m^2.7^ (*P* = 0.88), and LV mass-for-height z-score was −0.02 ± 1.13 vs. −0.02 ± 1.26 (*P* = 0.99).

### Cross-sectional comparison of those with and without LVH at baseline and at last follow-up

At baseline, there was no difference in hypertension prevalence between those with and without LVH by LVMI, with a higher rate of hypertension in the LVH group versus no LVH at follow-up (87.5% vs. 50% (*P* = 0.06)). Those with LVH had higher 24-h MAP at both baseline (96.4 ± 15.6 vs. 88.7 ± 11.7 mmHg, *P* = 0.032) and follow-up (100.1 ± 16.8 vs. 88.8 mmHg ± 7.7, *P* = 0.004) compared to those without LVH (Table 2).

**Table 2:** Clinical characteristics of those with and without left ventricular hypertrophy, defined using age-range based criteria at baseline and follow-up [18]

	LVH at baseline ***N*** = 12 (37.5%)	No LVH at baseline ***N*** = 20 (62.5%)	***P***	LVH at follow-up ***N*** = 8 (25%)	No LVH at follow-up ***N*** = 24 (75%)	***P***
Age at ABPM	12.4 ± 3.72	13.6 ± 2.9	0.32	13.4 ± 2.8	14.3 ± 3.3	0.52
Sex, female, *n* (%)	9 (75%)	5 (25%)	**0.01**	5 (62.5%)	9 (37.5%)	0.25
Glomerular disease, *n* (%)	6 (50%)	5 (25%)	0.15	4 (50%)	7 (29.2%)	0.28
Dialysis modality, IHD, *n* (%)	9 (75%)	8 (40%)	0.078	3 (37.5%)	14 (58.3%)	0.42
Antihypertensives, *n* = yes (% total)	10 (83.3%)	14 (70.0%)	0.68	8 (100%)	14 (58.3%)	**0.035**
**Office BP**						
SBP (mmHg)	121.9 ± 13.1	109.8 ± 12.3	**0.017**	122.3 ± 17.0	109.4 ± 11.6	**0.028**
SBP (z-score)	1.63 ± 1.44	0.17 ± 1.07	0.003	1.48 ± 1.38	0.08 ± 1.06	**0.007**
DBP (mmHg)	69.8 ± 11.6	65.9 ± 10.7	0.39	76.3 ± 16.0	71.1 ± 10.3	0.31
DBP (z-score)	0.70 ± 1.09	0.22 ± 0.87	0.23	1.22 ± 1.22	0.64 ± 0.87	0.17
Hypertensive on office BP, *n* (%)	4 (33.3%)	3 (15.0%)	0.17	3 (37.5%)	1 (4.2%)	**0.039**
**24—hr ABPM**						
MAP (mmHg)	96.4 ± 15.6	88.7 ± 11.7	**0.032**	100.1 ± 16.8	88.8 ± 7.7	**0.004**
MAP (z-score)	3.22 ± 3.30	1.21 ± 2.58	0.065	4.34 ± 4.34	0.76 ± 1.50	**0.006**
SBP (mmHg)	125.0 ± 15.7	116.9 ± 14.1	0.14	130.6 ± 19.5	114.9 ± 9.5	**0.005**
SBP (z-score)	1.75 ± 1.85	0.08 ± 1.81	**0.018**	2.06 ± 2.3	−0.09 ± 1.27	**0.002**
DBP (mmHg)	82.3 ± 16.4	74.7 ± 12.3	0.14	85.3 ± 17.8	72.5 ± 8.1	**0.009**
DBP (z-score)	3.02 ± 3.35	1.32 ± 2.30	0.098	3.60 ± 3.62	0.91 ± 1.56	**0.006**
Hypertensive on ABPM, *n* (%)	8 (66.6%)	11 (55.0%)	0.71	7 (87.5%)	12 (50%)	0.06
Masked hypertension, *n* (%)	5 (41.7%)	9 (45%)	0.85	4 (50%)	10 (41.7%)	0.68
**LV Function**						
Fractional shortening (FS) (%)	36.6 ± 5.7%	37.9 ± 6.3%	0.54	38.4 ± 5.6%	36.5 ± 5.4%	0.26
E/e’	8.70 ± 1.77	8.36 ± 2.34	0.68	9.38 ± 2.21	7.61 ± 2.67	0.11
E/e’ > 8.0, *n*/total (%)	7/11 (63.6%)	9/20 (45%)	0.46	6/8 (75%)	6/21 (28.6%)	**0.038**
Hb (g/l)	86 ± 11	104 ± 13	** < 0.001**	107 ± 14	113 ± 10	0.19
Log_10_ NT Pro-BNP (log pg/ml)	3.34 ± 0.60	2.85 ± 0.40	**0.011**	3.35 ± 0.51	2.72 ± 0.43	**0.003**

At baseline, there was no difference in the proportion of CYP on at least one anti-hypertensive (AHT) medication between groups, though a higher proportion of CYP with LVH at follow-up were prescribed AHTs (100% vs. 58.3%, *P* = 0.035). Twelve of 23 (52.2%) CYP prescribed AHTs were on more than one agent at baseline vs. 16/22 (72.73%) at follow-up (*P* = 0.15). All CYP on at least one AHT were prescribed the dihydropyridine calcium channel blocker (CCB) amlodipine at baseline, with all but one (95.5%) prescribed at least amlodipine at follow-up. The commonest second agents were alpha and beta blockers, with one CYP prescribed an angiotensin-converting enzyme (ACE) inhibitor and one an angiotensin receptor blocker.

Patients with LVH had lower haemoglobin at baseline (86 ± 11 vs. 104 ± 13 g/l, *P* < 0.001) and higher log-transformed NT Pro-BNP concentration at both baseline (3.34 ± 0.60 vs. 2.85 ± 0.40, *P* = 0.011) and follow-up (3.35 ± 0.51 vs. 2.72 ± 0.43, *P* = 0.003) compared with no LVH. Other laboratory parameters showed no significant differences except for higher phosphate in those with LVH at follow-up (2.1 ± 0.6 vs. 1.6 ± 0.3, *P* = 0.009) (Supplementary Table 2). See Supplementary Table 3 for further biochemical and body composition parameters of each blood pressure trajectory group and Supplementary Table 4 for the above characteristics within the changing blood pressure trajectory groups.

### Comparison of patients in the four blood pressure trajectory groups

Data comparing key parameters at baseline and follow-up in unchanged BP trajectory groups is presented in Table 3. Fourteen CYP (43.8%) had persistent hypertension, and eight (25%) had persistent normotension. In persistently hypertensive CYP, masked hypertension was present in 12/14 (85.7%) CYP at baseline and 10/14 (71.4%) at follow-up. In the emergent hypertension group, 4/5 (80%) CYP had masked hypertension at follow-up. There was no significant difference in the proportion of CYP with a glomerular primary kidney disease between groups (persistently hypertensive 6/14 (42.9%), persistently normotensive 2/8 (25%) *P* = 0.67).

**Table 3:** Cardiac, blood pressure, and selected biochemical characteristics within unchanging blood pressure trajectory groups

	Persistent hypertension ***N*** = 14			Persistent normotension ***N*** = 8		
Sex, female, *n* (%)	10 (71.4%)			3 (37.5%)		
Dialysis modality, IHD, *n* (%)	7 (50%)			3 (37.5%)		
Glomerular disease, *n* (%)	6 (42.9%)			2 (25%)		
	**Baseline**	**Follow-up**	***P***	**Baseline**	**Follow-up**	***P***
Age at ABPM	13.8 ± 2.8	14.7 ± 2.7	n/a	11.6 ± 3.7	12.4 ± 3.9	n/a
Antihypertensives, *n* = yes (%)				3 (37.5%)	3 (37.5%)	1
**LV structure and function**						
LVH by LVMI, *n* (%)	6 (42.9%)	6 (37.5%)	0.68	3 (37.5%)	1 (12.5%)	0.25
LVMI (g/m^2.7^)	44.9 ± 15.7	43.9 ± 17.9	078	36.0 ± 9.8	32.8 ± 6.8	0.14
LV Mass for height z-score	0.88 ± 1.51	0.74 ± 1.63	0.72	− 0.15 ± 1.26	− 0.59 ± 1.03	0.11
Fractional shortening (FS) (%)	37.8 ± 6.0	36.2 ± 5.7	0.42	36.6 ± 3.2	36.4 ± 3.6	0.85
E/e’	8.9 ± 1.9	8.8 ± 3.2	0.83	8.7 ± 3.4	6.8 ± 1.1	0.14
E/e’ > 8.0, *n* (% total)	9/14 (64.3%)	6/14 (42.3%)	0.37	3/6 (50%)	1/6 (16.7%)	0.48
**Office BP**						
Clinic SBP (mmHg)	118.7 ± 13.6	121.8 ± 12.0	0.45	105.1 ± 11.0	100.4 ± 8.3	0.41
Clinic SBP z-score	1.05 ± 1.29	1.25 ± 1.11	0.67	0.13 ± 1.21	− 0.46 ± 0.95	0.26
Clinic DBP (mmHg)	71.1 ± 14.1	77.6 ± 12.4	0.18	64.0 ± 5.0	65.6 ± 9.7	0.80
Clinic DBP z-score	0.63 ± 1.26	1.19 ± 1.05	0.23	0.26 ± 0.45	0.32 ± 0.84	0.96
Hypertensive on office BP, n (%)	2 (14.3%)	4 (25%)	0.48	1 (12.5%)	0 (0%)	1
**24—hr ABPM**						
MAP (mmHg)	101.4 ± 11.1	97.1 ± 13.3	0.21	77.3 ± 5.0	80.0 ± 4.3	0.12
MAP (z-score)	4.20 ± 2.94	3.31 ± 3.57	0.29	− 0.66 ± 0.82	− 0.28 ± 0.65	0.21
SBP (mmHg)	130.5 ± 11.6	127.3 ± 15.1	0.46	104.1 ± 8.5	107.0 ± 7.0	0.15
SBP z-score	2.18 ± 1.49	1.60 ± 1.87	0.31	− 1.06 ± 1.20	− 0.79 ± 0.82	0.28
DBP (mmHg)	87.6 ± 12.2	82.7 ± 13.9	0.14	62.6 ± 4.2	64.9 ± 3.7	0.15
DBP z-score	3.92 ± 2.58	2.96 ± 2.86	0.17	− 0.83 ± 0.75	− 0.42 ± 0.64	0.16
Masked hypertension, *n* (%)	12 (85.7%)	10 (71.4%)	0.48	0 (0%)	0 (0%)	1
Hb (g/l)	91.6 ± 12.9	108.1 ± 11.8	**0.003**	102.8 ± 8.8	112.4 ± 11.9	0.18
Log_10_ NT Pro-BNP (log pg/ml)	3.13 ± 0.56	3.05 ± 0.56	0.63	2.91 ± 0.41	2.84 ± 0.57	0.73

**Fig. 1:**
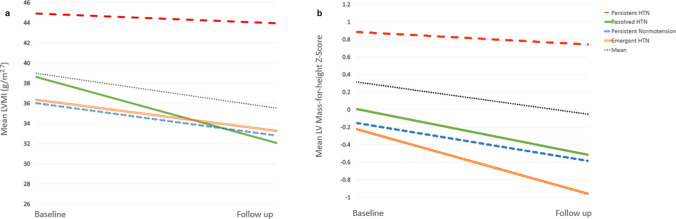
**a**) and **b**) Mean LVMI and mass-for-height z-score between baseline and follow-up by blood pressure trajectory group

Indexed LV mass and LV mass-for-height z-score decreased across all groups with the greatest LVMI reduction in the resolved hypertension group (Fig. 1a and b). There was no significant difference in LVM between persistently hypertensive and persistent normotensive groups at baseline (LVMI 44.9 ± 15.7 vs. 36.0 ± 9.8 (*P* = 0.13) and LV mass-for-height z-score 0.88 ± 1.51 vs. −0.15 ± 1.26 (*P* = 0.10)), but at follow-up, differences widened (LVMI 43.9 ± 17.9 vs. 32.8 ± 6.8 g/m^2.7^ (*P* = 0.05) and LV mass-for-height z-score 0.74 ± 1.63 vs. −0.58 ± 1.03 (*P* = 0.03)).

In the persistent hypertension group, seven children (50%) had an increase in indexed LV mass with a median (range) ΔLVMI of 7.09 g/m^2.7^ (2.4–10.3) and ΔLV mass-for-height z-score 1.21 (0.60–1.29). In those with persistent normotension, two children (25%) had an increase in LVMI of 1.74 and 2.19 g/m^2.7^ and LV mass-for-height z-score of 0.41 and 0.30. Persistently hypertensive CYP had lower Hb at baseline compared with persistently normotensive CYP (91.6 ± 12.9 vs. 102.8 ± 8.8 g/l (*P* = 0.027)), with no difference at follow-up.

### Left ventricular systolic and diastolic function

A higher proportion of CYP with an E/e’ > 8 was found in those with LVH versus without at follow-up (75% vs. 28.6%, *P* = 0.038). Only one child in the entire cohort, without LVH at baseline, had an E/e’ measure > 14, at 15.1.

### Determinants of ΔLVMI and risk of developing LVH

**Fig. 2:**
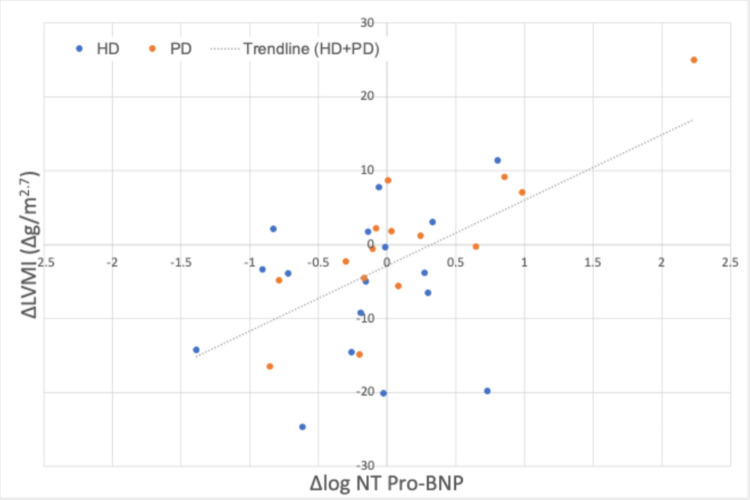
Scatterplot of Δlog NT Pro-BNP against ΔLVMI (Δg/m.^2.7^) shown by dialysis modality (*R* = 0.584, *P* < 0.001)

Univariate regression analysis revealed a significant relationship between ΔLVMI and Δlog NT Pro-BNP (*R* = 0.584, *P* < 0.001) (see Fig. 2), ΔHb (*R *= 0.540, *P* = 0.001), LVH at baseline (*R* = 0.629, *P *< 0.001), and ΔOffice SBP (*R* = 0.457, *P* = 0.009). Of all ABPM readings (night, day, and 24 h), only ΔNight SBP (*R* = 0.418, *P* = 0.017) correlated significantly with ΔLVMI.

Following multivariable regression analyses, LVH at baseline (yes/no) (*β* = −0.482, *P* = 0.001) and Δlog NT Pro-BNP (*β* = 0.412, *P* = 0.005) remained significant independent predictors of ΔLVMI (adjusted r^2^ 0.512, *P* = < 0.001, variance inflation factor 1.146). There were no changes to the final model when Δ24-h SBP and Δ24-h DBP were substituted for Δ24-h MAP in the regression model.

## Discussion

In this report, we present data to highlight the relevance of managing hypertension optimally in CYP receiving dialysis. Following commencement of dialysis, those with persistent hypertension had the highest risk of LVH and increased indexed LV mass compared with those CYP who were persistently normotensive.

Previous longitudinal studies report varied relationships between BP and HMOD indices in CYP receiving dialysis [3–5, 13]. Ulinski et al. demonstrated a reduction in indexed LV mass correlating with improved office BP in 17 CYP on IHD [5]. Mitsnefes et al. reported no significant changes to indexed LV mass in 29 CYP after a mean 10 months on IHD, though they did report correlation between indexed BP and indexed LV mass [3, 4]. In a report from our centre, Melhem et al. found improved BP control as the most important factor associated with LV remodelling in 29 CYP commencing IHD for a median 21 months [13]. Our study expands on these reports including patients on peritoneal dialysis and haemodialysis and defining hypertension precisely using ABPM.

The relevance of ABPM for diagnosing hypertension is underscored with masked hypertension at 43.8% both at baseline and follow-up and markedly higher in those with persistent hypertension at ~ 85%. This contrasts with a cross-sectional study of children with CKD reporting rates of 29.7% [16].

A range of other studies consistently demonstrate poor correlation between office and ambulatory BP. A meta-analysis of 692 adult patients on HD reported high variability between casual pre-dialysis SBP and interdialytic ambulatory SBP, with agreement limits of −25.2 to 41.7 mmHg [21]. In a cohort study of 24 CYP on HD, only ABPM day and night-time diastolic load correlated with LVH, with no correlation against post-dialysis casual BP [15]. Such data relays the importance of using ABPM to guide interventions, as reflected in recent UK guidelines recommending its use where possible [22].

The prevalence of LVH at baseline, at ~ 38%, was lower than previous studies reporting 69–82% [3, 5, 13]. This may reflect inclusion of patients on PD in whom the rate of LVH was lower. The prevalence of LVH improved in CYP following initiation of IHD, but not for those initiating CCPD, potentially reflecting the differential impact of dialysis modalities. As all patients underwent protocolised cardiovascular evaluation, it is less likely that any differences reflect divergences in the surveillance of CYP on IHD versus CCPD, although we collected no data to consider this precisely.

We attempted to evaluate ‘fluid overload’, a widely accepted driver of hypertension in CYP on dialysis. Previous studies highlight a significant positive association between LVMI and a median IDWD of ≥4% in 16 CYP receiving HD [23] and of carotid intima media thickness and IDWD ≥4% in a study of 19 CYP receiving HD [24]. In our study, we found no similar association of IDWD with hypertension or LVH. We were also unable to precisely obtain data regarding urine output. These discrepancies highlight difficulties in the accurate assessment of dry weight in CYP, an ongoing challenge given dynamic shifts in fluid status on dialysis alongside changes to growth, nutrition, and body composition.

Our study is the first of its kind to evaluate NT Pro-BNP and its association with LVH. Recent work has shown that BNP is associated with kidney damage but also a potential marker of fluid status in CYP receiving haemodialysis [25, 26]. We extend these observations in a clinically meaningful way observing that those with persistent hypertension had higher NT Pro-BNP concentrations compared to persistently normotensive CYP. We found higher NT Pro-BNP concentrations in those with LVH compared to no LVH at baseline and follow-up. Further, Δlog NT pro-BNP was found to correlate with ΔLVMI. Taken together, these data suggest that in CYP on dialysis with persistent hypertension and increased NT Pro-BNP, fluid overload is likely with a higher chance of associated HMOD. In these patients specifically, renewed efforts to reduce fluid overload should be implemented. These findings are hypothesis generating, and more research is indicated aiming to develop more precise recommendations around interpretation and clinical utility of NT Pro-BNP.

The overall antihypertensive medication burden was similar at both timepoints, though a higher proportion of patients with LVH versus those without LVH were on AHTs at follow-up. Almost all CYP in our study on at least one AHT were on amlodipine. At present, no clear evidence exists to recommend any agents over others in CYP receiving dialysis, though dialysability and pharmacokinetics should be taken into consideration when choosing a suitable agent [22]. In a US survey, ACE inhibitors and dihydropyridine CCBs were most frequently prescribed [27]. A registry study of CYP receiving PD and a prospective study of CYP with CKD both suggest a reduced risk of LVH with renin–angiotensin system antagonists against other agents [28, 29]. Ramipril has been shown to reduce markers of endothelial dysfunction in CYP receiving HD, potentially conferring additional cardiovascular benefits beyond reducing BP [30]. Further longitudinal studies assessing the cardiovascular impacts of different agents are warranted.

Haemoglobin levels were lower in those with LVH at baseline but not at follow-up, and in keeping with previous studies demonstrating an independent association between anaemia and LVH in kidney failure [3, 4, 31]. Our observation of a stronger association between hypertension and LVH at follow-up demonstrates that uncontrolled hypertension plays a greater role in the persistence of LVH on dialysis.

Extending our previous report [13], we included E/e’ to provide an insight regarding differences in diastolic function within our cohort. In contrast to other HMOD indices, abnormal diastolic function is an early reported feature of CKD [32–35] and is associated with heart failure with preserved ejection fraction in CYP and adults [35, 36]. Larger cross-sectional studies also suggest an independent association between worse E/e’ and BP [37, 38], with proposed mechanisms including changes to ventricular relaxation and changes to blood volume [35, 39].

Our cut-off E/e’ value of > 14.0 denoting clinically significant elevated LV filling pressure concurs with international recommendations for adults [20], though common to other studies we found that few children reached this threshold [11, 40]. In CYP, E/e’ is affected by age [41], and an algorithm for diagnosing clinically significant diastolic dysfunction in children with E/e’ in the 8.0–14.0 “indeterminate” range has not been established [11]. An exploratory analysis of diastolic function in CYP with CKD in the HOT-KID cohort included a wider range of measures (E/A, early to late diastolic LV filling velocity ratio; LAVi, body surface area indexed left atrial volume; e’/a’, early to late diastolic peak mitral annular velocity ratio) which may provide an instructive blueprint to future studies appraising diastolic function in CYP. UK adult guidelines recommend including left atrial volume and tricuspid regurgitation velocity as well as E/e’ and measures of left atrial strain within an algorithm to determine diastolic dysfunction, although we accept this may not be directly transferable to CYP [42]. We demonstrate a higher proportion of CYP with E/e’ > 8 in those with LVH versus without at follow-up, though the clinical significance of this remains unclear.

Recent evaluations of the 4 C and HOT-KID cohorts suggest that systolic function can be impaired earlier than previously thought in childhood CKD [2, 10, 43]. As with diastolic function, there is evidence of a relationship between BP control and systolic function independent of LV structure [44]. In our study, no clear associations between systolic function and BP control were demonstrated. A recent evaluation of first-phase ejection fraction (EF1) found it to be a more sensitive marker of systolic dysfunction over FS [10], and its inclusion in future studies would be welcome.

Limitations to our study included its single centre, small numbers, and retrospective nature. There was a varied timespan between baseline and follow-up, and our delta estimates may have been affected by this. Future studies may benefit from analysing ΔLVMI against Δlog NT pro-BNP over a more precisely defined timeframe. Despite this, patients underwent protocolised evaluations and were managed by a dedicated team of professionals. We accept that our study may have benefitted from the inclusion of data on sodium intake, loss, and regarding sodium dialysate and serum sodium as further measures to evaluate fluid overload, and future studies would benefit from its inclusion. Future studies would also benefit from replicating protocols in larger populations allowing for assessment of the impact of underlying kidney disease, dialysis modality, and residual kidney function. Our cohort included CYP from varied ethnic backgrounds, yet while normative values for LV structure and function differ between populations [44], our reporting does not account for this. Studies were conducted as routine clinical care, and we were unable to assess whether children were symptomatic at assessment. We could not assess compliance with fluid restrictions and anti-hypertensive medications.

## Conclusion

In this longitudinal cohort of CYP initiating dialysis, the most significant improvements in LV mass are seen in those with the greatest improvement in BP status. CYP on IHD demonstrated an improvement in rates of LVH between baseline and follow-up, but not in CYP on CCPD. NT Pro-BNP shows promise as a marker of fluid overload which may help tailor strategies when managing hypertension in those CYP receiving dialysis. A high rate of masked hypertension supports the use of ABPM to guide intervention.

## Supplementary Information

Below is the link to the electronic supplementary material.

Graphical abstract (PPTX 808 KB)

ESM 1  (DOCX 221 KB)

## Data Availability

The datasets generated during and/or analysed during the current study are available from the corresponding author on reasonable request.
