# Supplementary material for: Hypertension control and its association with left ventricular hypertrophy in children and young people on dialysis: a longitudinal study
Source: Pediatr Nephrol. 2026 May 23;41(10):3595–604. doi: 10.1007/s00467-026-07351-1 (PMC13481484; doi:10.1007/s00467-026-07351-1)
Supplement: Supplementary file 1 [file 467_2026_7351_MOESM2_ESM.docx]

**Supplementary Information**

**Article Title:** Hypertension control and its association with left ventricular hypertrophy in children and young people on dialysis: a longitudinal study

**Authors**: Gordon Bruce^1^, Ravsimrat Chawla^1^, Alex Savis^2^, Hannah Samuels^1^, Joanna Newton^1^, John M Simpson^2^, Manish D Sinha PhD ^1,3*^

**Affiliations**^: 1^Department of Paediatric Nephrology, Evelina London Children’s Hospital, Guys & St Thomas NHS Foundation Trust, Westminster Bridge Road, London SE1 7EH; ^2^Department of Paediatric Nephrology, Evelina London Children’s Hospital, Guys & St Thomas NHS Foundation Trust, Westminster Bridge Road, London SE1 7EH; ^3^Department of Clinical Pharmacology, School of Cardiovascular and Metabolic Medicine, King’s College London

^*^**Corresponding author**: Professor Manish D Sinha, Department of Paediatric Nephrology, 3^rd^ Floor Beckett House, Evelina London Children’s Hospital, Guys & St Thomas NHS Foundation Trust, Westminster Bridge Road, LONDON SE1 7EH, United Kingdom.

Tel: +44 20 7188 4587

Email: [manish.2.sinha@kcl.ac.uk](mailto:manish.2.sinha@kcl.ac.uk)

|  | | Haemodialysis (n=17) | | | Peritoneal Dialysis (n=15) | | | | P |
| --- | --- | --- | --- | --- | --- | --- | --- | --- | --- |
| Age at first ABPM | | 13.6 ± 3.5 | | | 12.7 ± 2.9 | | | | 0.43 |
| Sex, female, n (%) | | 8 (47.1%) | | | 6 (40.0%) | | | | 0.69 |
| Glomerular disease, n (%) | | 7 (41.1%) | | | 4 (26.7%) | | | | 0.39 |
|  | | | | | | | | | |
|  | | **Baseline** | | |  | **Follow Up** | | |  |
|  | | **Haemodialysis (n=17)** | | **Peritoneal Dialysis (n=15)** | **P** | **Haemodialysis (n=17)** | | **Peritoneal Dialysis (n=15)** | **P** |
| LV Structure and function | | | | | | | | | |
|  | LVH by LVMI, n (%) | 9 (52.9%) | 3(20%) | | 0.06 | 3 (17.6%) | 5 (33.3%) | | 0.31 |
|  | LVMI (g/m^2.7^) | 43.7 ± 16.2 | 36.1 ± 7.5 | | 0.11 | 37.8 ± 17.4 | 36.5 ± 9.5 | | 0.79 |
|  | LV Mass for height z-score | 0.61 ± 1.73 | -0.02 ± 1.13 | | 0.24 | -0.08 ± 1.77 | -0.02 ± 1.26 | | 0.90 |
|  | Fractional shortening (FS) (%) | 35.2 ± 5.9 | 39.7 ± 5.5 | | **0.04** | 37.3 ± 5.3 | 36.7 ± 5.8 | | 0.73 |
|  | E/e’ | 8.9 ± 2.5 | 8.0 ± 1.5 | | 0.27 | 8.4 ± 2.9 | 7.7 ± 2.2 | | 0.45 |
|  | E/ e’ >8.0, n (% total) | 10 (58.8%) | 6 (40%) | | 0.29 | 4 (23.5%) | 4 (26.7%) | | 0.84 |
| Hypertension | | | | | | | | |  |
| Hypertensive on Office BP, n (%) | | 6 (35.3%) | 1 (6.7%) | | 0.05 | 6 (35.3%) | 2 (13.3%) | | 0.15 |
| Hypertensive on ABPM, n (%) | | 12 (70.6%) | 7 (46.7%) | | 0.17 | 9 (52.9%) | 10 (66.7%) | | 0.43 |
| Masked HTN, n (%) | | 7 (41.1%) | 7 (46.7%) | | 0.75 | 5 (29.4%) | 9 (60%) | | 0.08 |
| Antihypertensives, n=yes (% total) | | 15 (88.2%) | 10 (66.7%) | | 0.14 | 11 (64.7%) | 11 (73.3%) | | 0.60 |
| Biochemical parameters | | |  | | | | | | |
| Hb (g/l) | | 92.5 ± 16.6 | 102.2 ± 10.9 | | 0.06 | 113.5 ± 9.4 | 109.8 ± 13.4 | | 0.37 |
| Albumin (g/l) | | 40.9 ± 3.2 | 40.8 ± 7.0 | | 0.94 | 39.9 ± 10.3 | 40.1 ± 2.8 | | 0.94 |
| Phosphate (mmol/l) | | 1.52 ± 0.31 | 1.75 ± 0.35 | | 0.06 | 1.65 ± 0.52 | 1.79 ± 0.34 | | 0.39 |
| Adjusted Calcium (mmol/l) | | 2.40 ± 0.13 | 2.43 ± 0.12 | | 0.46 | 2.45 ± 0.11 | 2.53 ± 0.14 | | 0.11 |
| PTH (pg/ml) | | 272.4 ± 222.1 | 335.5 ± 270.7 | | 0.47 | 316.8 ± 421.6 | 285.5 ± 245.6 | | 0.80 |
| Log_10_ NT Pro-BNP (log pg/ml) | | 3.21 ± 0.6 | 2.85 ± 0.38 | | 0.07 | 2.82 ± 0.46 | 2.92 ± 0.60 | | 0.62 |

**Table 1:** Comparison between children and young people on haemodialysis vs peritoneal dialysis including characteristics and baseline vs follow up.

|  | LVH at baseline  N = 12 (37.5%) | No LVH at baseline  N = 20 (62.5%) | P | LVH at follow up  N = 8 (25%) | No LVH at follow up  N = 24 (75%) | P |
| --- | --- | --- | --- | --- | --- | --- |
| IDWD (%) | 0.05 ± 0.5 (9/12) | 0.65 ± 2.1(8/20) | 0.45 | 0.02 ± 0.02 (3/8) | 0.61 ± 1.70 (14/23) | 0.64 |
| Albumin (g/l) | 39.5 ± 5.03 | 41.7 ± 5.34 | 0.26 | 40.8 ± 4.3 | 39.8 ± 8.5 | 0.76 |
| Phosphate (mmol/l) | 1.54 ± 0.35 | 1.68 ± 0.34 | 0.29 | 2.1 ± 0.6 | 1.6 ± 0.3 | **0.009** |
| Adjusted Calcium (mmol/l) | 2.37 ± 0.15 | 2.44 ± 0.10 | 0.15 | 2.54 ± 0.12 | 2.47 ± 0.13 | 0.19 |
| PTH (pg/ml) | 306.6 ± 260.3 | 299.2 ± 240.8 | 0.94 | 348.2 ± 362.0 | 286.8 ± 346.1 | 0.67 |

**Table 2**: Additional biochemical and body composition parameters of those with and without LVH, defined using age-range based criteria at baseline and follow up [18].

|  | Persistent Hypertension  N = 14 | | | Resolved Hypertension  N = 5 | | | | Persistent Normotension  N = 8 | | | *Emergent Hypertension  N = 5 | | |
| --- | --- | --- | --- | --- | --- | --- | --- | --- | --- | --- | --- | --- | --- |
|  | **Baseline** | **Follow up** | **p** | **Baseline** | **Follow up** | **p** | **Baseline** | | **Follow up** | **p** | **Baseline** | **Follow up** | **p** |
| IDWD (%) | 1.59 ± 1.9 (7/14) | 1.32 ± 1.1 |  | 0.24 ± 1.7 (5/5) | 1.10 ± 1.03 | 0.33 | 0.09 ± 1.26 (2/8) | | -0.65 ± 2.65 |  | -0.05 ± 0.17 (3/5) | -2.6 ± 0 (1/5) |  |
| Albumin (g/l) | 39.1 ± 5.2 | 40.8 ± 3.5 | 0.30 | 40.8 ± 3.5 | 41.2 ± 7.0 | 0.86 | 44.8 ± 2.05 | | 41.4 ± 3.5 | **0.018** | 39.8 ± 7.9 | 40.0 ± 3.4 | **0.035** |
| Phosphate (mmol/l) | 1.64 ± 0.41 | 1.80 ± 0.47 | 0.23 | 1.55 ± 0.32 | 1.46 ± 0.13 | 0.60 | 1.73 ± 0.28 | | 1.86 ± 0.46 | 0.48 | 1.5 ± 0.25 | 1.5 ± 0.43 | 1 |
| Adjusted Calcium (mmol/l) | 2.43 ± 0.13 | 2.45 ± 0.12 | 0.66 | 2.34 ± 0.15 | 2.42 ± 0.11 | 0.20 | 2.45 ± 0.12 | | 2.59 ± 0.12 | 0.05 | 2.4 ± 0.1 | 2.5 ±0.12 | 0.30 |
| PTH (pg/ml) | 401.5 ± 258.6 | 432.9 ±465.7 | 0.79 | 175.4 ± 136.5 | 90.5 ± 49.8 | 0.21 | 288.3 ± 272.8 | | 282.3 ± 185.9 | 0.94 | 171.7 ± 139.3 | 179.4 ± 143.3 | 0.83 |

**Table 3**: Biochemical and body composition parameters of each blood pressure trajectory group. *Emergent hypertension (i.e. those with newly occurred hypertension) had normotension at baseline and hypertension at follow up.

|  | | | | Resolved Hypertension  N = 5 | | | | | *Emergent Hypertension  N = 5 | | | |
| --- | --- | --- | --- | --- | --- | --- | --- | --- | --- | --- | --- | --- |
| Sex, female, n (%) | | | | 0 (0%) | | | | | 1(20%) | | | |
| Dialysis modality, IHD, n (%) | | | | 5 (100%) | | | | | 2 (40%) | | | |
| Glomerular disease, n (%) | | | | 2 (40.0%) | | | | | 1 (20%) | | | |
|  | | | | **Baseline** | | **Follow up** | **p** | | **Baseline** | **Follow up** | | **p** |
| Age at ABPM | | | | 13.1 ± 4.0 | | 14.1 ± 4.0 | n/a | | 13.9 ± 2.9 | 14.8 ± 2.8 | | n/a |
| Antihypertensives, n=yes(%) | | | | 4 (80%) | | 3 (60%) | 1.0 | | 4 (80%) | 3 (60%) | | 0.49 |
| LV structure and function | | | |  | |  |  | |  |  | |  |
|  | | LVH by LVMI, n (%) | | 2 (40%) | | 0 (0%) | 0.48 | | 1 (20%) | 0 (0%) | | 0.29 |
|  | | LVMI (g/m^2.7^) | | 38.6 ± 12.1 | | 32.1 ± 3.6 | 0.32 | | 36.3 ± 7.9 | 33.3 ± 8.5 | | 0.50 |
|  | | LV Mass for height z-score | | 0.005 ± 1.59 | | -0.52 ± 0.65 | 0.58 | | -0.20 ± 1.54 | -0.96 ±1.76 | | 0.20 |
|  | | Fractional shortening (FS) (%) | | 34.5 ± 9.2 | | 35.4 ± 5.2 | 0.87 | | 40.6 ± 5.9 | 42.0 ± 6.3 | | 0.70 |
|  | | E/e’ | | 8.0 ± 2.1 | | 7.5 ± 2.3 | 0.69 | | 7.8 ± 0.25 | 8.4 ± 2.2 | | 0.63 |
|  | | E/ e’ >8.0, n (% total) | | 2/5 (40%) | | 3/5 (60%) | 1.0 | | 1/4 (25%) | 3/4 (75%) | | 0.16 |
| Office BP | | |  | |  | |  |  | | |  | |
|  | Clinic SBP (mmHg) | | | 119.8 ± 14.1 | | 102.8 ± 8.7 | **0.03** | | 108.3 ± 9.5 | 116.4 ± 10.2 | | 0.82 |
|  | Clinic SBP z-score | | | 1.13 ± 1.69 | | -0.57 ±0.95 | **0.02** | | -0.10 ± 1.70 | 0.51 ± 0.85 | | 0.98 |
|  | Clinic DBP (mmHg) | | | 62.4 ± 8.9 | | 70.0 ± 10.1 | 0.38 | | 70.0 ± 10.0 | 71.2 ± 10.3 | | 0.59 |
|  | Clinic DBP z-score | | | 0.004 ± 1.0 | | 0.55 ± 0.67 | 0.43 | | 0.58 ± 0.54 | 0.62 ± 1.00 | | 0.55 |
| Hypertensive on office BP, n (%) | | | | 2 (40%) | | 0 (0%) | 0.48 | | 1 (20%) | 1 (20%) | | 1 |
| 24-hr ABPM | | | |  | |  |  | |  |  | |  |
|  | | MAP (mmHg) | | 96.6 ± 8.9 | | 83.4 ± 4.7 | **0.027** | | 82.0 ± 4.7 | 93.8 ±4.3 | | **0.021** |
|  | | MAP (z-score) | | 2.04 ± 1.44 | | 0.03 ± 0.89 | **0.02** | | -0.15 ± 0.96 | 1.75 ±1.38 | | **0.025** |
|  | | SBP (mmHg) | | 125.2 ± 9.8 | | 108.2 ± 4.3 | **0.03** | | 110.2 ± 7.0 | 124.8 ± 3.6 | | **0.004** |
|  | | SBP z-score | | 0.98 ± 1.31 | | -1.06 ± 1.00 | **0.01** | | -0.84 ± 1.30 | 0.73 ± 1.17 | | **0.012** |
|  | | DBP (mmHg) | | 83.4 ± 8.3 | | 69.4 ± 5.9 | **0.02** | | 67.4 ± 4.3 | 79.4 ± 5.9 | | **0.027** |
|  | | DBP z-score | | 2.91 ± 1.61 | | 0.31± 1.19 | **0.02** | | -0.05 ± 0.92 | 2.21 ± 1.39 | | **0.029** |
| Masked Hypertension, n (%) | | | | 2 (40%) | | 0 | 0.48 | | 0 | 4 (80%) | | 0.13 |
| Hb (g/l) | | | | 96.6 ± 25.3 | | 115.6 ± 10.2 | 0.27 | | 103.6 ± 12.9 | 117.2 ± 10.4 | | 0.12 |
| Log_10_ NT Pro-BNP (log pg/ml) | | | | 3.16 ± 0.69 | | 2.66 ± 0.33 | 0.15 | | 2.84 ± 0.57 | 2.53 ± | | 0.18 |

**Table 4:** Cardiac, blood pressure and selected biochemical characteristics within the changing blood pressure trajectory groups. *Emergent hypertension (i.e. those with newly occurred hypertension) had normotension at baseline and hypertension at follow up.
